# A randomized-controlled trial of community-based transdiagnostic psychotherapy for veterans and internally displaced persons in Ukraine

**DOI:** 10.1017/gmh.2021.27

**Published:** 2021-08-27

**Authors:** Sergiy Bogdanov, Jura Augustinavicius, Judith K. Bass, Kristie Metz, Stephanie Skavenski, Namrita S. Singh, Quincy Moore, Emily E. Haroz, Jeremy Kane, Ben Doty, Laura Murray, Paul Bolton

**Affiliations:** 1 Center for Mental Health and Psychosocial Support, National University of Kyiv-Mohyla Academy, Kyiv, Ukraine; 2 Department of Mental Health, Johns Hopkins Bloomberg School of Public Health, Baltimore, USA; 3 Department of International Health, Johns Hopkins Bloomberg School of Public Health, Baltimore, USA; 4 Department of Epidemiology, Columbia University Mailman School of Public Health, New York, USA

**Keywords:** CETA, Brief CETA, community-based, trial, mental health, military conflict, psychotherapy

## Abstract

**Background:**

There is limited research on community-based mental health interventions in former Soviet countries despite different contextual factors from where most research has been conducted. Ongoing military conflict has resulted in many displaced persons and veterans and their families with high burdens of mental health problems. Lack of community-based services and poor uptake of existing psychiatric services led to the current trial to determine the effectiveness of the common elements treatment approach (CETA) on anxiety, depression, and posttraumatic stress symptoms (PTS) among conflict affected adults in Ukraine.

**Methods:**

We conducted a three-armed randomized-controlled trial of CETA delivered in its standard form (8–12 sessions), a brief form (five-sessions), and a wait-control condition. Eligible participants were displaced adults, army veterans and their adult family members with elevated depression and/or PTS and impaired functioning. Treatment was delivered by community-based providers trained in both standard and brief CETA. Outcome data were collected monthly.

**Results:**

There were 302 trial participants (*n* = 117 brief CETA, *n* = 129 standard CETA, *n* = 56 wait-controls). Compared with wait-controls, participants in standard and brief CETA experienced clinically and statistically significant reductions in depression, anxiety, and PTS and dysfunction (effect sizes *d =* 0.46–1.0–6). Comparing those who received standard CETA with brief CETA, the former reported fewer symptoms and less dysfunction with small-to-medium effect sized (*d* = 0.20–0.55).

**Conclusions:**

Standard CETA is more effective than brief CETA, but brief CETA also had significant effects compared with wait-controls. Given demonstrated effectiveness, CETA could be scaled up as an effective community-based approach.

## Introduction

Since 2014 military conflict with Russia-backed separatists has left 13 000 casualties and 3.4 million people requiring humanitarian assistance (United Nations Office for the Coordination of Humanitarian Affairs, 2020). Conflict affected populations in Ukraine have high prevalence rates for post-traumatic stress disorder (PTSD; 32%), depression (22%), anxiety (17%) and alcohol use disorders (8.4% in men and 0.7% in women) (Weissbecker *et al*., 2017; Ramachandran *et al*., 2019; Roberts *et al*., 2019). 74% of adult internally displaced persons (IDPs) who likely required mental health services reported not receiving them due to lack of trust in the health system, lack of awareness of where to seek care, poor quality of services, stigma and embarrassment, and/or logistical barriers (e.g. no local clinics or providers, disrupted roadways, etc.) (Weissbecker *et al*., 2017).

This has occurred on top of longstanding service issues. The mental health system in Ukraine remains highly centralized with most services provided by psychiatrists in psychiatric clinics and hospitals. Since the 1960s Ukrainians have shown great reluctance to seek help at inpatient services because of the stigma associated with admission to psychiatric hospitals and reports of abuse under the Soviet system, as well as health system deficiencies like lack of information and awareness, high cost of treatment, fear of a public record of mental illness diagnosis, and geographical distance (Weissbecker *et al*., 2017; Romaniuk and Semigina, 2018). The prevalence of common mental health problems in the general population has increased over the past decade (Voloshyn and Maruta, 2015; Weissbecker *et al*., 2017; Ramachandran *et al*., 2019; Roberts *et al*., 2019) while the prevalence of clinician diagnoses and treatment has decreased (Voloshyn and Maruta, 2015), likely due to ongoing stigmatization of psychiatric services (Weissbecker *et al*., 2017) and budgetary and economic issues (Skokauskas *et al*., 2020).The burden of common mental health problems would likely be better met by mental health care at the community level. Elsewhere, community-based mental health treatment models have been developed to reduce the treatment gap (Kohn *et al*., 2004; Thornicroft, 2007; Thornicroft *et al*., 2016) by making services more culturally responsive and locally accessible (Castillo *et al*., 2019), can treat multiple problems while being less resource intensive than specialized care, and are therefore more scalable and sustainable (World Health Organization, 2017; Patel *et al*., 2018).

The common elements treatment approach (CETA) (Murray *et al*., 2014*a*), a cognitive-behavioral therapy-based treatment, was developed as a community-based intervention to address multiple and comorbid mental health problems in low- and middle-income countries (LMIC) (Murray *et al*., 2014*a*, *2*020). While CETA has been shown effective in other LMIC settings including Iraq (Weiss *et al*., 2015), Thailand (Bolton *et al*., 2014), Zambia (Murray *et al*., 2020), Colombia (Bonilla-Escobar *et al*., 2018) and Ethiopia (Murray *et al*., 2018*b*), it has not been used or tested in the former Soviet Union. This is true of the global mental health literature overall: most LMIC intervention trials, both clinic- and community-based, have been conducted in Africa with few in Asia and Latin America (Chibanda *et al*., 2015; Purgato *et al*., 2018). In our review of the English-language literature, we found no trials from former Soviet Union countries, where cultural and contextual factors are very different from other regions. The few trials in the Russian language literature found locally modified cognitive-behavioral psychotherapy effective for anxiety disorders in a general psychiatric population (Tukaev and Kuznetsov, 2015) and motivational interviewing effective for alcohol use disorders (Trusova, 2015). Given the contextual and cultural differences that set this region apart from other global regions, we felt it necessary to test CETA locally.

We also explored a key implementation question: whether a shortened five-session version of CETA could be as effective as the standard 8–12 session model in order to address scale-up challenges, particularly with highly mobile populations, including displaced persons.

To address these aims, this study had two objectives: (1) to evaluate the effectiveness of standard CETA among a conflict-affected population in Ukraine; and (2) to compare the effectiveness of brief CETA to standard CETA using a non-inferiority design.

## Methods

### Study design

This study is a three-armed randomized-controlled trial conducted among a conflict-affected population: adult IDPs and Ukrainian veterans and their families. The three conditions included: (1) standard CETA, (2) brief CETA, and (3) waitlist controls; participants in the CETA arms were blind to their allocation to brief or standard versions. The primary outcomes of the trial included severity of depression and post-traumatic stress symptoms, and impaired functioning and secondary outcomes included anxiety symptoms and substance use problems. This trial is registered with ClinicalTrials.gov NCT03058302.

### Providers

The trial was conducted in Kyiv (central region), Zaporizhya (southeast), and Kharkiv (east, near Russian border). Thirty-one individuals working in these communities were trained as providers by CETA co-developers (Laura K. Murray, s.d.) and other CETA trainers (Stephanie Skavenski, Kristie Metz, Laura Merchant) using the apprenticeship model (Murray *et al*., 2011) of didactic training followed by supervised practice. Concurrently, five others were trained as local supervisors. Providers were psychologists, social workers, volunteers, physicians, program managers, teachers/lecturers or lawyers (Murray *et al*., 2018*a*, 2018*b*). Some were experienced serving IDPs and other conflict-affected groups and some were veterans. Prior qualitative research found that veterans are more willing to talk to other veterans (Singh *et al*., 2021). Fifteen providers were based in Kyiv City, four in Kharkiv City, eight in Zaporizhya City, and four were in smaller towns in Zaporizhya region. Providers met with participants individually in private rooms, typically at the provider's place of employment, state social services, or local non-governmental organizations (NGOs). No sessions were conducted in hospitals or state mental health institutions. Recruitment materials and the CETA manual for provider were offered in Ukrainian language while the implementation and the mental health assessment inventory (MHAI) outcome measure, a previously validated tool to assess common mental health problems (Doty *et al*., 2018), were in Russian language because the majority of study participants, mostly IDPs, preferred Russian.

### Participants

Participants had to give informed consent, be at least 18 years old, and expect to remain in a study location for at least 6 months and be either a veteran, veteran family member, or IDP. We also included people who volunteered in the conflict or were otherwise helping affected persons. These population criteria were based on the target population of our funder. Clinical inclusion criteria included reporting elevated depression and/or posttraumatic stress symptoms (PTS) and impaired daily functioning at baseline. Screening for elevated symptoms and impaired functioning was done using a shortened version of the outcome instrument (MHAI; described below) (Doty *et al*., 2018).

Exclusion criteria included active-duty (non-veteran) military personnel and adults who were determined at baseline to need urgent referral to a psychiatrist for suicidal or homicidal ideation or severe psychiatric symptoms or drug use problems that necessitate inpatient treatment and would inhibit their ability to participate in a talk-therapy. These adults were determined by local clinical CETA supervisors and psychiatrist at the National University of Kyiv-Mohyla Academy (NaUKMA) Mental Health Center. Adults excluded based on these criteria were referred to external specialist mental health services.

Study participants were referred by providers from their existing work-related network and by community-based organizations (CBOs) and NGOs working with these populations. CBO and NGO staff were trained to use a short screening version of the outcome measure (Doty *et al*., 2018) and refer potential participants who showed PTSD or depression symptoms.

### Outcome measure

The outcome measure, the MHAI, was locally adapted and validated to assess clinically significant mental health problems (Doty *et al*., 2018). The MHAI includes items related to the primary and secondary common mental health outcomes (depression, posttraumatic stress, generalized anxiety, and alcohol use) and a functioning scale. The validation process is described elsewhere (Doty *et al*., 2018). The functioning scale includes World Health Organization Disability Assessment Schedule (WHODAS) items and locally relevant items based on a prior qualitative study in Zaporizhya and Kharkiv (Applied Mental Health Research Group Johns Hopkins University, 2013; Singh *et al*., 2021).

### Sample size

For comparison of brief and standard CETA to the waitlist control condition, the sample size was based on power of 0.80 (*β* = 0.20) at *α* = 0.025 level of significance (adjusted for multiple comparisons), to detect a medium effect (*f* = 0.25). Sample size for the non-inferiority comparison of brief v. standard CETA was based on power of 0.80 (*β* = 0.20) at *α* = 0.05 level of significance, with an assumed s.d. of 7.0 and a non-inferiority limit of *d* = 2.4. Accounting for 24% attrition, the recruitment goal was 294 participants.

### Randomization

We implemented a two-stage sequential randomization process. First stage randomization was conducted by computer algorithm into waitlist control or CETA conditions (brief and standard combined) at a 1:4 ratio. The algorithm was integrated into the data collection software with randomization immediately after determining trial eligibility. The second stage allocated those in the CETA condition to either brief or standard treatment; randomization was based on a computer-generated list of random numbers with a ratio of 1:1. This second stage randomization was done in blocks of 20 by provider, to ensure evenly distribution among providers.

### Blinding

First stage randomization (waitlist control/CETA) was not blinded but revealed to participants immediately after baseline assessment. For second stage randomization, participants, providers, supervisors, and CETA trainers were kept blind to allocation to brief or standard CETA until after the fourth CETA session. The study director then checked assignment and revealed the allocation to the supervisor and provider who then told the study participant. Only the study director and two analysts on the data monitoring team had access to the password-protected database linking study ID to the brief or standard CETA randomization schema. This ensured that randomization to brief or standard CETA did not impact the way the first four sessions were provided by provider or received by participant.

### Interventions

Two version of CETA – standard and brief – were tested; (for full descriptions see trial protocol manuscript (Murray *et al*., 2018*a*, 2018*b*). CETA is a modular, multi-problem, flexible, transdiagnostic approach developed for LMIC, based on task-sharing by providers with minimal or no formal mental health training (Murray *et al*., 2014*a*, 2018*a*, 2018*b*). We designed the brief CETA model based on recent research on how to increase efficiency without losing effectiveness (Hayes *et al*., 2007*a*, 2007*b*; Huebner and Tonigan, 2007; Insel, 2009).

Participants randomized to standard CETA began with the same first session flows as brief CETA. Providers had flexibility in choosing the elements, order and dose for participants in standard CETA beyond the fourth session, based on symptom presentation and discussions of participant progress with their supervisors (Murray *et al*., 2014*a*).

Throughout the trial, supervision followed the apprenticeship model (Murray *et al*., 2011) with weekly provider oversight by CETA trainers to monitor and ensure treatment fidelity for both standard and brief CETA. Both versions of CETA also included safety check ins at every session (Murray *et al*., 2014*b*). All safety cases were handled within 24-hours with immediate notification of NaUKMA and Johns Hopkins School of Public Health (JHSPH) research teams.

### Waitlist control condition

Waitlist control participants were provided with a list of available mental health resources and, like all trial participants, were free to use these services. All use of other services was tracked over the course of the trial. After participating in the waitlist control condition for 6 months, control participants were offered standard CETA.

### Data collection

Data were collected via a self-administered digital survey using the open source mobile data collection platform CommCare (https://www.commcarehq.org). Study enrollment and data collection began in March 2017 and data collection was completed in June 2019. Baseline assessments (time 0) were completed prior to first stage CETA/waitlist control randomization. After baseline, waitlist control participants were asked to complete monthly assessments until 6-months post-baseline (times 1–6). CETA participants also completed these assessments after every fourth weekly visit with their counselor. After completion of standard or brief CETA treatment, participants continued to complete assessments every month until the end of the 6 month period. Because treatment participants may not be able to attend the CETA sessions every week, for brief CETA participants, we expected 3–4 of these assessments would be completed after treatment was over and for standard CETA participants, we expected 2–3 of these assessments would be completed after treatment was over. Baseline and follow-up assessments were done at a central location in each city using study tablets, with study staff present to support the technology and answer questions. In cases where a study participant could not attend in person study staff would conduct the assessment by phone.

During baseline screening, 73 clients reported suicidal or homicidal ideations, triggering the safety protocols. Most were found to be at low risk after additional questioning by study M&E staff; 17 were referred to a psychiatrist for further assessment. Although they were not included in the study, standard CETA was offered to all of these clients after evaluation. During the study, there were no adverse events.

At the beginning of clinical sessions, as a normal part of treatment, a clinical monitoring form was self-administered on CommCare to guide treatment. This trial was reviewed and approved by the JHSPH and the NaUKMA Institutional Review Boards.

### Statistical analysis

As per the trial protocol brief and standard CETA were each compared separately to the waitlist control condition using the six monthly assessments collected post baseline (Murray *et al*., 2018*a*, 2018*b*). Due to variations in participants availability for the assessment we included assessments up to 2 weeks prior or after the targeted monthly date. Baseline assessments were coded as assessment zero (0). Each subsequent monthly assessment was coded as assessment 1, 2, through 6. Under the protocol analysis we planned to investigate the effects of brief and standard CETA, compared with the waitlist control condition over these six discrete timepoints. Figure 1*a* presents the flow of participants included in the protocol analysis.

**Fig. 1. (a):**
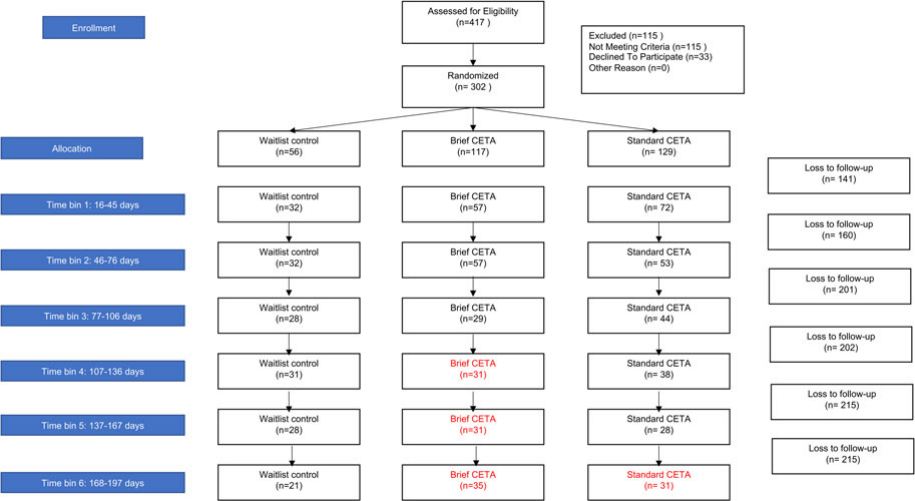
Consort diagram for flow of participants through monthly assessments (plus or minus 2 weeks) as per trial protocol.

In reviewing the data at the end of the trial, we identified that some CETA participants did not complete treatment within the 6-month time frame or had only one post-treatment assessments, providing none or limited post-treatment assessment data making the planned longitudinal analyses untenable for these participants. Therefore, a post-hoc analysis approach was also used, to provide a more equivalent comparison between study arms. Using a pre-post design, we used a single post-intervention assessment for each brief and standard CETA participant and an equivalent assessment for each waitlist control participant. To remain in line with the original planned analysis of outcomes at 6 months post baseline, for this analysis we used each participant's post-intervention assessment closest to the 6 month follow up (180 days plus 122 days or minus 30 days). The amount of change was calculated for each participant by comparing this single post-treatment assessment to their baseline. Figure 1*b* presents the flow of participants included in the pre-post analysis.

**Fig. 1. (b):**
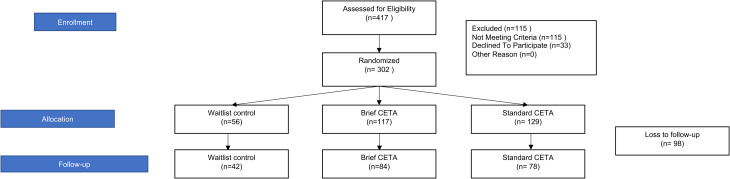
Flow of participants through pre-post assessments as relevant to the post-hoc analysis.

All outcomes (depression, anxiety, and PTS, and functioning) were treated as continuous. Sample characteristics were explored across treatment conditions at baseline. Random effects models with a robust variance estimator were used to compare change in outcomes between trial conditions. Sensitivity analyses examined the impact on model results of including the number of days on study (between baseline and the final assessment) as a covariate. For the second analysis, multiple imputation with chained equations accounted for scale level missingness of outcome data (Azur *et al*., 2011). Using imputed data, the effects of brief and standard CETA on symptoms and functioning between baseline and follow-up were assessed using mixed effects regression models with clustering at the individual, provider, and city levels. Cohen's *d* was used to calculate effect sizes for each outcome, reflecting regression adjustments. Statistical analyses were performed using Stata 15 (Stata Statistical Software, 2017).

## Results

The study sample (Table 1) included 181 (59.9%) women and 121 (40.1%) men. Participants lived in Kyiv (69%), Kharkiv (8%), or Zaporizhya (23%) during the study period. Participants identified as IDPs (40%), veterans (32%), family members of veterans (22%), or volunteers in the conflict (15%). Most had completed some post-secondary education or training (68%) and 65% had attended university.

**Table 1.** Baseline characteristics of brief, standard and waitlist control participants

	Brief CETA (*n* = 117)	Standard CETA (*n* = 129)	Waitlist control (*n* = 56)
Mean age in years, mean (s.d.)	38.75 (10.45)	39.25 (10.48)	39.05 (10.46)
Female, no. (%)	72 (62)	74 (57)	35 (63)
Location, no. (%)			
Kyiv	85 (73)	90 (70)	32 (57)
Kharkiv	10 (9)	9 (7)	6 (11)
Zaporizhya	22 (19)	30 (23)	18 (32)
Status, no. (%)			
IDP	46 (39)	51 (40)	24 (43)
Veteran	38 (32)	42 (33)	16 (29)
Veteran family member	26 (22)	28 (22)	12 (21)
Conflict volunteer	21 (18)	18 (14)	5 (9)
Marital status, no. (%)			
Single	25 (22)	33 (26)	18 (33)
Married	48 (41)	58 (45)	24 (44)
Widowed	10 (9)	11 (9)	2 (4)
Divorced	33 (28)	26 (20)	11 (20)
Employment, no. (%)			
Unemployed	22 (19)	22 (17)	11 (20)
Self-employed	10 (9)	5 (4)	2 (4)
Occasional job	30 (26)	31 (24)	13 (23)
Formal job	55 (47)	71 (55)	30 (54)
Education, no. (%)			
None	1 (1)	–	–
Primary	(2)	–	3 (5)
Secondary	14 (12)	13 (10)	8 (14)
Tech/professional	25 (21)	25 (19)	7 (13)
University	72 (62)	88 (68)	36 (64)
Post-graduate	3 (3)	3 (2)	2 (4)
Mean depression score, mean (s.d.)	1.51 (0.55)	1.58 (0.59)	1.53 (0.56)
Mean posttraumatic stress score, mean (s.d.)	1.39 (0.58)	1.49 (0.58)	1.30 (0.62)
Mean anxiety score, mean (s.d.)	1.37 (0.60)	1.37 (0.61)	1.31 (0.60)
Mean dysfunction score, mean (s.d.)	1.23 (0.58)	1.29 (0.67)	1.17 (0.66)

NB, Symptom scores for depression, posttraumatic stress, anxiety, and dysfunction represent mean item scores.

Chi square and *t* test comparisons of demographic characteristics between treatment conditions at baseline did not reveal any significant differences except for mean post-traumatic stress symptoms where there was a marginally significant difference in mean scores between standard CETA and waitlist control groups *t*(183) *=* −1.99, *p* = 0.05.

Baseline assessments were completed by 302 participants across the trial conditions (brief CETA *n* = 117, standard CETA *n* = 129, waitlist control = 56) (see Consort diagram Fig. 1*a*). Only nine participants (3%) had data for all six-monthly assessments after baseline. Sixty-four participants (21%), had no data after baseline [11 (20%) control, 21 (18%) brief CETA, and 32 (25%) standard CETA]; 108 participants (36%) had data from only one or two of the monthly assessments. Seven CETA participants (six standard; one brief) were still receiving treatment at the 6-month post baseline assessment time point, so this final assessment was not post-treatment.

The planned protocol analysis, showing overall trends for predicted symptom and dysfunction scores by treatment condition over the 6-month follow-up period, are shown in Supplement Figures 1S–4S. The results of unimputed models indicated that brief and standard CETA participants had statistically significantly lower predicted depression, post-traumatic stress, anxiety, and dysfunction scores on at least one time point during follow up relative to waitlist controls. Standard CETA participants had statistically significantly lower average predicted depression and posttraumatic stress scores at least one time point during follow up relative to brief CETA participants. Sensitivity analyses, including the number of days on study, did not change the direction or significantly alter the magnitude of treatment effects.

For the pre-post analysis, we used data from the 204 participants with post-intervention (brief and standard CETA participants) or post-baseline (waitlist control participants) assessment data. The included assessment data were conducted an average of 211 days after baseline (range of 150–299 days). Follow up data were missing from 98 participants [brief CETA *n* = 33 (28%), standard CETA *n* = 51 (40%), waitlist control = 14 (25%)].

Treatment effects using this pre-post analysis for brief and standard CETA compared with waitlist controls are presented in Tables 2 and treatment effects comparing brief to standard CETA are presented in Table 3. Reductions in depression, post-traumatic stress, anxiety, and dysfunction were greater among those receiving standard CETA relative to those in the waitlist control condition with medium to large effect sizes (*d* = 0.60–1.06). Reductions in all outcomes were also observed among those receiving brief CETA, relative to waitlist controls with medium effect sizes (*d* = 0.46–0.62).

**Table 2.** Pre-post changes in study outcomes for brief and standard CETA compared to Waitlist controls

Outcomes	Brief CETA	Standard CETA	Waitlist control
Depression			
Baseline, mean (s.e.)	1.48 (0.06)	1.56 (0.08)	1.51 (0.11)
Follow-up, mean (s.e.)	0.67 (0.06)	0.43 (0.07)	1.00 (0.06)
Pre-post change	−0.81 (−0.87 to −0.76)	−1.12 (−1.21 to −1.04)	−0.51 (−0.71 to −0.31)
Net effect (*β*, 95% CI)*	−0.30 (−0.45 to −0.15)	−0.61 (−0.73 to −0.50)	–
Effect estimate (*d*)	0.55	1.06	–
Posttraumatic stress			
Baseline, mean (s.e.)	1.38 (0.07)	1.48 (0.05)	1.30 (0.07)
Follow-up, mean (s.e.)	0.61 (0.09)	0.44 (0.07)	0.88 (0.09)
Pre-post change	−0.77 (−0.88 to −0.66)	−1.04 (−1.15 to −0.93)	−0.42 (−0.58 to −0.26)
Net effect (*β*, 95% CI)*	−0.35 (−0.46 to −0.25)	−0.62 (−0.82 to −0.43)	–
Effect estimate (*d*)	0.59	1.05	–
Anxiety			
Baseline, mean (s.e.)	1.36 (0.03)	1.36 (0.04)	1.31 (0.12)
Follow-up, mean (s.e.)	0.68 (0.04)	0.49 (0.08)	1.00 (0.08)
Pre-post change	−0.68 (0.04)	−0.87 (0.05)	−0.31 (0.13)
Net effect (*β*, 95% CI)*	−0.37 (−0.57 to −0.17)	−0.56 (−0.90 to −0.22)	–
Effect estimate (*d*)	0.62	0.93	–
Dysfunction			
Baseline, mean (s.e.)	1.18 (0.02)	1.26 (0.07)	1.14 (0.09)
Follow-up, mean (s.e.)	0.84 (0.09)	0.79 (0.12)	1.07 (0.20)
Pre-post change	−0.35 (0.07)	−0.47 (0.06)	−0.07 (0.13)
Net effect (*β*, 95% CI)*	−0.28 (−0.42 to −0.14)	−0.40 (−0.57 to −0.24)	–
Effect estimate (*d*)	0.46	0.60	–

NB, Means are from predicted models and take into account clustering and imputation.

*This is the interaction term beta and 95% CI.

**Table 3.** Pre-post changes in study outcomes for standard CETA compared to brief CETA

Outcomes	Standard CETA
Depression	
Net effect (*β*, 95% CI)	−0.31 (−0.36 to −0.27)
Effect estimate (*d*)	0.55
Posttraumatic stress	
Net effect (*β*, 95% CI)	−0.27 (−0.48 to −0.06)
Effect estimate (*d*)	0.47
Anxiety	
Net effect (*β*, 95% CI)	−0.19 (−0.34 to −0.04)
Effect estimate (*d*)	0.32
Dysfunction	
Net effect (*β*, 95% CI)	−0.12 (−0.16 to −0.09)
Effect estimate (*d*)	0.20

NB, Means are from predicted models and take into account clustering and imputation.

When comparing the effectiveness of standard to brief CETA, those receiving standard CETA reported fewer symptoms and less dysfunction at post-assessment, with medium effect sizes for depression (*d* = 0.55) and post-traumatic stress symptoms (*d* = 0.47) and small effect sizes for anxiety symptoms (*d* = 0.32) and dysfunction (*d* = 0.20). When comparing imputed to non-imputed models in sensitivity analyses, only minimal differences were observed between effect estimates (see Online Supplemental tables 1S and 2S).

Among waitlist control participants, only seven reported seeking outside mental health services. These consisted of an appointment with a psychiatrist in a mental health clinic or in a general polyclinic.

## Discussion

This trial examined the effectiveness of both standard and brief CETA compared to a wait-list control condition among veterans and IDPs in three areas of Ukraine during a period of instability and conflict. Both brief and standard CETA reduced the severity of depression, anxiety and PTS and functional impairment, with the strongest impacts for those who participated in standard CETA.

Among possible community-based interventions CETA was chosen due to the comorbidity we found within populations of veterans and IDPs in our qualitative work (Singh *et al*., 2021), similar to findings in other trauma/conflict-affected populations. CETA is a particular approach – namely modular, flexible and multi-problem (see more on transdiagnostic definitions (Boustani *et al*., 2017; Sauer-Zavala *et al*., 2017; Martin *et al*., 2018) able to address stress and traumatic events including conflict and deprivation, *and* common comorbidities - depression, anxiety, violence, suicide, and substance use. Use of a more limited transdiagnostic approach such as Problem Management Plus (PM+) (which does not include a direct focus on traumatic stress symptoms) was therefore not considered appropriate for this type population (Dawson *et al*., 2015).

To date we have tested CETA in Africa, southeast Asia, South America, and the Middle East. While Ukraine is culturally part of Europe where similar interventions have been tested, the combination of differences between eastern and western Europe, the strong historical legacy of the former Soviet Union, and the dearth of effectiveness studies of Mental Health and Psychosocial Support (MHPSS) interventions in the former Soviet region suggested a need for local testing.

That standard CETA had a greater impact on symptom reduction compared with brief CETA could suggest a dose response effect of more sessions or additional elements. The standard condition allowed for more personalization and time to address multiple problems. It also provides more contact with the provider. Other possibilities include contextual and population-based factors. Throughout the trial, many participants or their family and friends were traveling to the Russian front, re-living battles, hearing about them, and experiencing stresses related to conflict (e.g. losing loved ones, losing homes). This ongoing context may require longer treatment. It would be interesting to evaluate a shorter version of CETA in a less traumatic or stressful context, or perhaps with a population that presented with milder symptomatology.

Many study participants in the brief CETA condition were unhappy about ending treatment, as were CETA providers, if they thought a participant needed more treatment. This could be partly due to cultural expectations of therapy being longer and needing to be longer. To a lesser degree, some study participants and CETA providers were unhappy about being ‘forced’ to continue with treatment after their symptoms greatly reduced.

Participants in both standard and brief CETA reported significant and similar effects on function compared to waitlist controls. This suggests that CETA may have improved function through mechanisms other than symptom reduction and independently of CETA duration. Perhaps a connection to a counselor and/or the presence of certain elements were important to improving function but not the dose of elements.

### Limitations

Missing participant follow-up assessments were a significant limitation of the trial. The originally trial analysis plan expected that CETA participants would attend treatment sessions weekly and that monthly data collection would occur after every fourth treatment visit. Often treatment visits did not happen every week and so the duration between data collection points was not consistent across CETA participants and some CETA participant remained in treatment for more than 6 months. This rendered the results of our original analysis plan as suggestive only, in that many of the planned monthly assessments for standard CETA occurred while treatment was being provided, rather than some being completed during treatment and some after treatment completion. Some of the reasons treatment completion was delayed included lengthy breaks and holidays, trips back to the conflict areas, changing living area, and/or successful employment that resulted in lack of time for attending sessions. These delays and dropouts produced a reduced sample of follow up data biased towards those participants who were more invested and compliant with treatment. For this reason, we added a pre-post analysis that allowed us to include a less biased sample, although missing data were still a limitation. These challenges were greater in this study than in previous trials in other global regions, partly due to local cultural (e.g. long holiday periods), and situational variables (e.g. ongoing conflict).

Causes of missed sessions and missed assessments among all participants were: (a) IDPs tended to move during the trial and to leave no means of further contact, and (b) participants often changed phone numbers without explanation or providing new numbers. Among the participants in the CETA conditions, additional factors included, (a) dissatisfaction about being provided with brief treatment (i.e. 4–5 sessions), and (b) annoyance at being contacted outside treatment. Continuing attempts of Monitoring and Evaluation (M&E) staff to follow up with participants was understood as a culturally inappropriate imposition and resulted in some participants rejecting all contacts with the research team. To address these concerns: (a) a recruitment officer reached out to family members and related peer groups and local communities to obtain new contact information/phone numbers of lost participants; (b) CETA counselors reached out to their participants and asked them to complete assessment forms; and (c) M&E staff asked participants who rejected participation in follow up assessments to complete the monthly assessment form at least once.

We had limited information on what treatments the waitlist control participants accessed beyond reporting whether they consulted a psychiatrist or psychologist in part due to a programming error with our data collection system which did not ask for details on the kind of help they received. For those participants who did provide information on whether they had sought out mental health services, we received only a few responses and almost no descriptions. It appears that the engagement with mental health services outside of CETA was very limited based on self-report.

Finally, while we provided substance use specific elements in both brief and standard CETA, we were not able to complete impact analyses on substance use outcomes due to an error in programming the data collection software. This error resulted in a lack of consistency in data collection on the substance use outcomes limiting the reliability of the data we do have. Therefore, those results are not reported.

## Conclusions

This study constitutes the first trial in Ukraine, and in the former Soviet region, of a community-based approach for common mental health problems. Despite study and program challenges, we found that standard CETA is more effective for reducing symptoms of common mental health problems than brief CETA, but brief CETA also had significant effects compared with waitlist controls. Both versions of CETA had a similar effect on function. Based on these results, the standard CETA model is preferred because it includes the full range of treatment elements and can be more appropriately tailored to the needs of each client. Brief CETA can be considered a reasonable option where the standard model is not possible due to logistical constraints, such as with highly mobile populations. Given the concerns of providers and participants that treatment was sometimes too long or too short, we recommend that CETA not be confined to ‘standard’ or ‘Short’. Rather, the end of treatment should be determined by the provider in consultation with the participant, based on improved symptoms, mastery of skills, and/or participant desires. As a modular approach that is designed to vary with client needs, CETA is suitable for such variations in duration. Future research should utilize implementation science designs to evaluate the uptake, effectiveness, and acceptability and cost of CETA of varying length in this way.

This study, building on the evidence from CETA trials, suggests that CETA should be expanded within Ukraine. With the advent of the coronavirus disease 2019 (COVID-19) pandemic we have developed distance-based tools and training materials for CETA, and have trained local providers who are now fully capable of acting as local trainers. These will result in capacity to conduct training, supervision, and service provision by internet and phone. This increased capacity will allow for expanding program reach throughout Ukraine, including to rural areas and border areas with ongoing instability, most of which currently have no mental health service access.

## Supplementary material

For supplementary material accompanying this paper visit http://dx.doi.org/10.1017/gmh.2021.27. click here to view supplementary material
