# Supplementary material for: A randomized-controlled trial of community-based transdiagnostic psychotherapy for veterans and internally displaced persons in Ukraine
Source: Glob Ment Health (Camb). 2021 Aug 27;8:e32. doi: 10.1017/gmh.2021.27 (PMC8392687; doi:10.1017/gmh.2021.27)
Supplement: Supplementary file 1 [file S2054425121000273sup001.docx]

**Supplementary Material**

Table 1S. Pre-post changes in study outcomes for Brief and Standard CETA compared to Waitlist controls (unimputed)

| Outcomes | | Brief CETA | Standard CETA | Waitlist Control |
| --- | --- | --- | --- | --- |
| Depression | |  |  |  |
|  | Baseline, mean (se) | 1.47 (0.07) | 1.58 (0.10) | 1.51 (0.11) |
|  | Follow-up, mean (se) | 0.66 (0.07) | 0.44 (0.08) | 1.00 (0.06) |
|  | Pre-post change | -0.81 (0.03) | -1.14 (0.04) | -0.51 (0.10) |
|  | Net effect ($\beta,$95% CI)* | -0.30 (-0.44, -0.16) | -0.63 (-0.75, -0.51) | - |
|  | Effect estimate (*d*) | 0.54 | 1.09 | - |
| Posttraumatic Stress | |  |  |  |
|  | Baseline, mean (se) | 1.37 (0.10) | 1.49 (0.06) | 1.30 (0.06) |
|  | Follow-up, mean (se) | 0.60 (0.10) | 0.43 (0.08) | 0.88 (0.09) |
|  | Pre-post change | -0.77 (0.06) | -1.05 (0.05) | -0.42 (0.08) |
|  | Net effect ($\beta,$95% CI)* | -0.35 (-0.49, -0.22) | -0.63 (-0.83, -0.44) | - |
|  | Effect estimate (*d*) | 0.59 | 1.06 | - |
| Anxiety | |  |  |  |
|  | Baseline, mean (se) | 1.35 (0.05) | 1.37 (0.07) | 1.31 (0.12) |
|  | Follow-up, mean (se) | 0.67 (0.06) | 0.49 (0.10) | 1.00 (0.09) |
|  | Pre-post change | -0.68 (0.04) | -0.89 (0.05) | -0.31 (0.13) |
|  | Net effect ($\beta,$95% CI)* | -0.37 (-0.55, -0.19) | -0.58 (-0.92, -0.24) | - |
|  | Effect estimate (*d*) | 0.62 | 0.95 | - |
| Dysfunction | |  |  |  |
|  | Baseline, mean (se) | 1.20 (0.01) | 1.25 (0.10) | 1.14 (0.09) |
|  | Follow-up, mean (se) | 0.83 (0.09) | 0.78 (0.13) | 1.07 (0.20) |
|  | Pre-post change | -0.36 (0.08) | -0.48 (0.03) | -0.07 (0.13) |
|  | Net effect ($\beta,$95% CI)* | -0.29 (-0.42, -0.17) | -0.41 (-0.61, -0.20) | - |
|  | Effect estimate (*d*) | 0.49 | 0.61 | - |

NB: Means are from predicted models and take into account clustering.

* This is the interaction term beta and 95% CI

Table 2s. Pre-post changes in study outcomes for Standard CETA compared to Brief CETA (unimputed)

| Outcomes | | Standard CETA |
| --- | --- | --- |
| Depression | |  |
|  | Net effect ($\beta,$95% CI) | -0.33 (-0.37, -0.30) |
|  | Effect estimate (*d*) | 0.59 |
| Posttraumatic Stress | |  |
|  | Net effect ($\beta,$95% CI) | -0.28 (-0.50, -0.07) |
|  | Effect estimate (*d*) | 0.49 |
| Anxiety | |  |
|  | Net effect ($\beta,$95% CI) | -0.21 (-0.37, -0.04) |
|  | Effect estimate (*d*) | -0.35 |
| Dysfunction | |  |
|  | Net effect ($\beta,$95% CI) | -0.11 (-0.21, -0.02) |
|  | Effect estimate (*d*) | -0.18 |

NB: Means are from predicted models and take into account clustering.


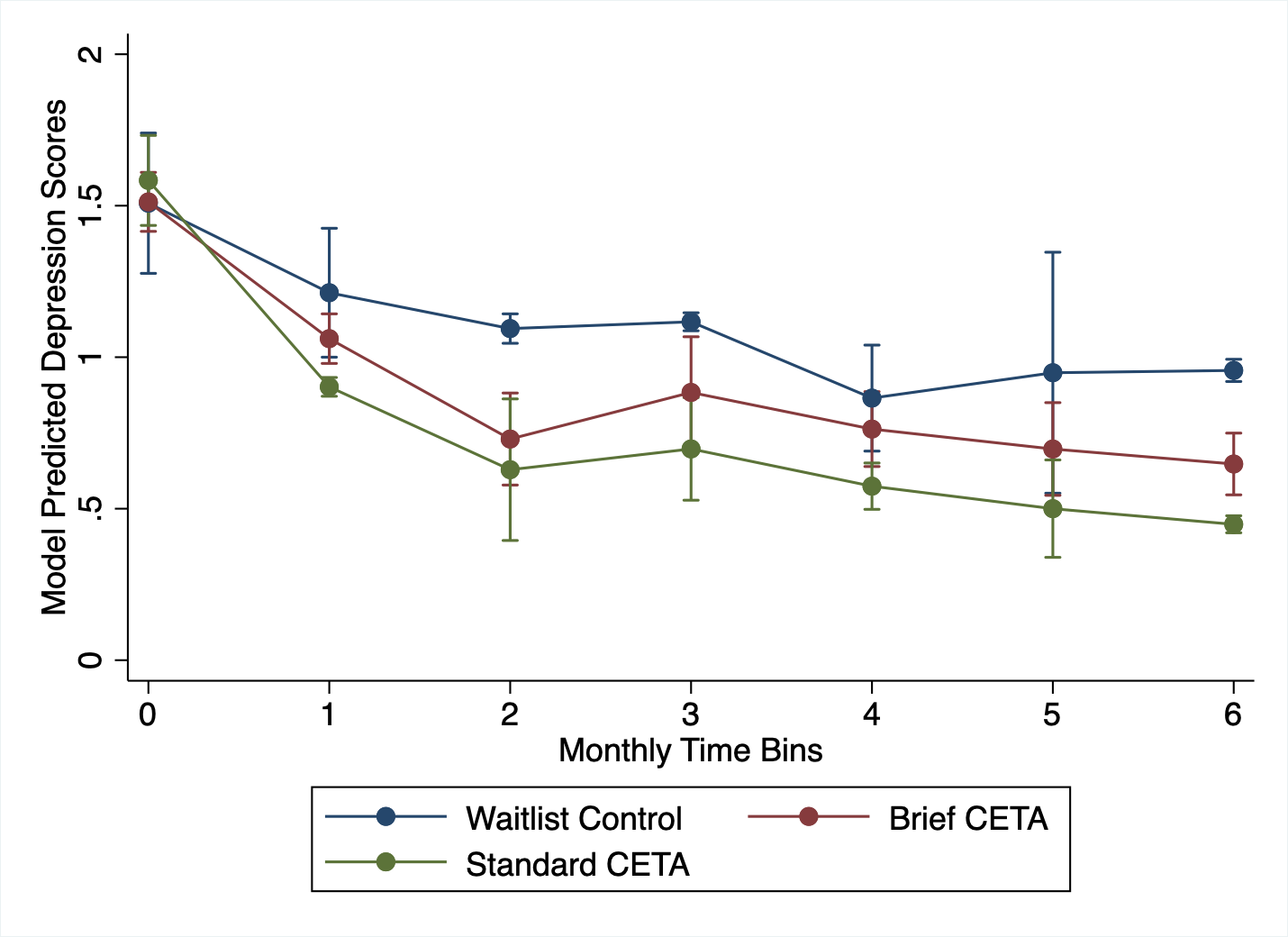


**Figure 1S.** Model predicted depression scores by treatment group over the six month period after baseline


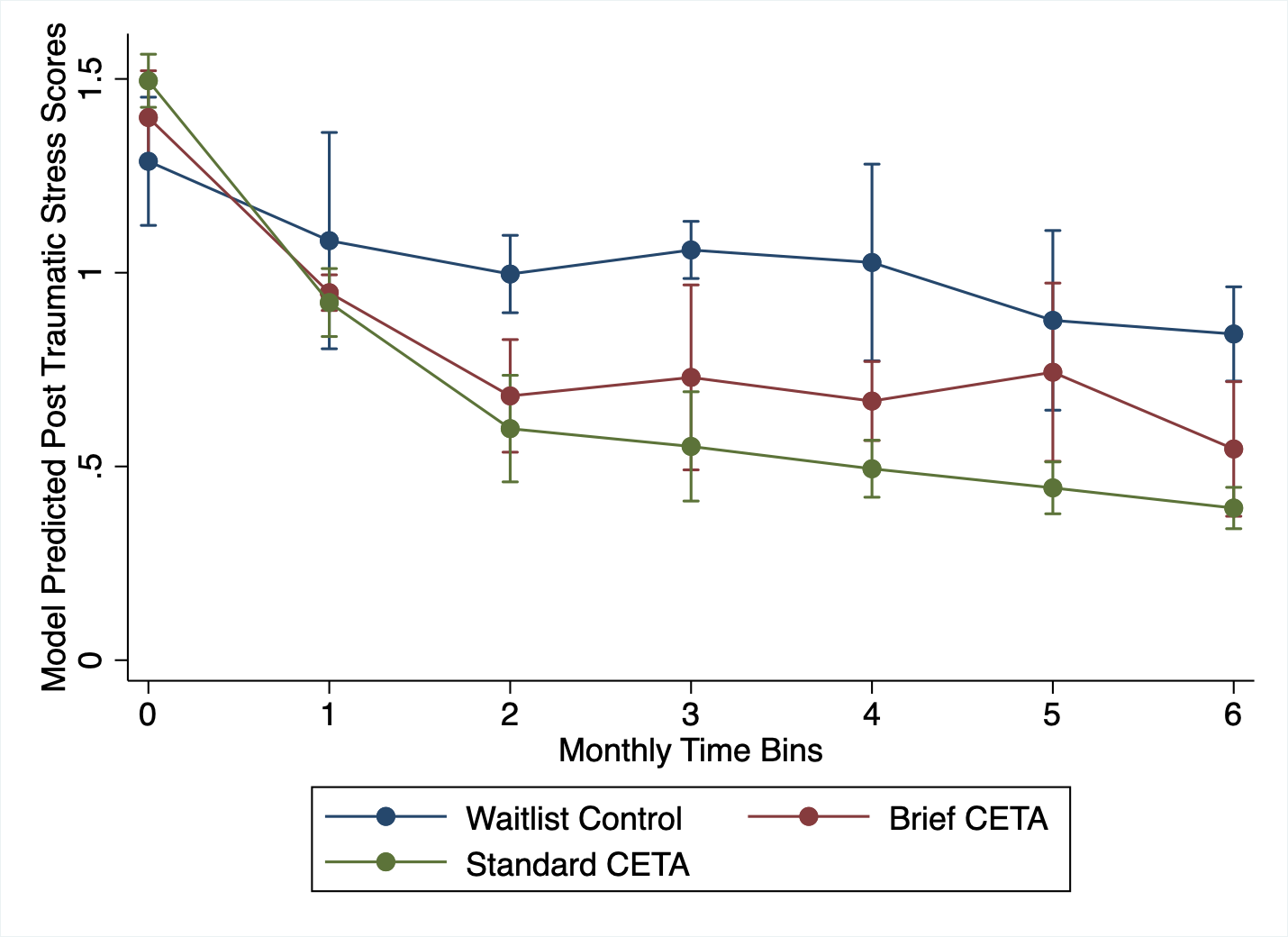


**Figure 2S.** Model predicted posttraumatic stress scores by treatment group over the six month period after baseline


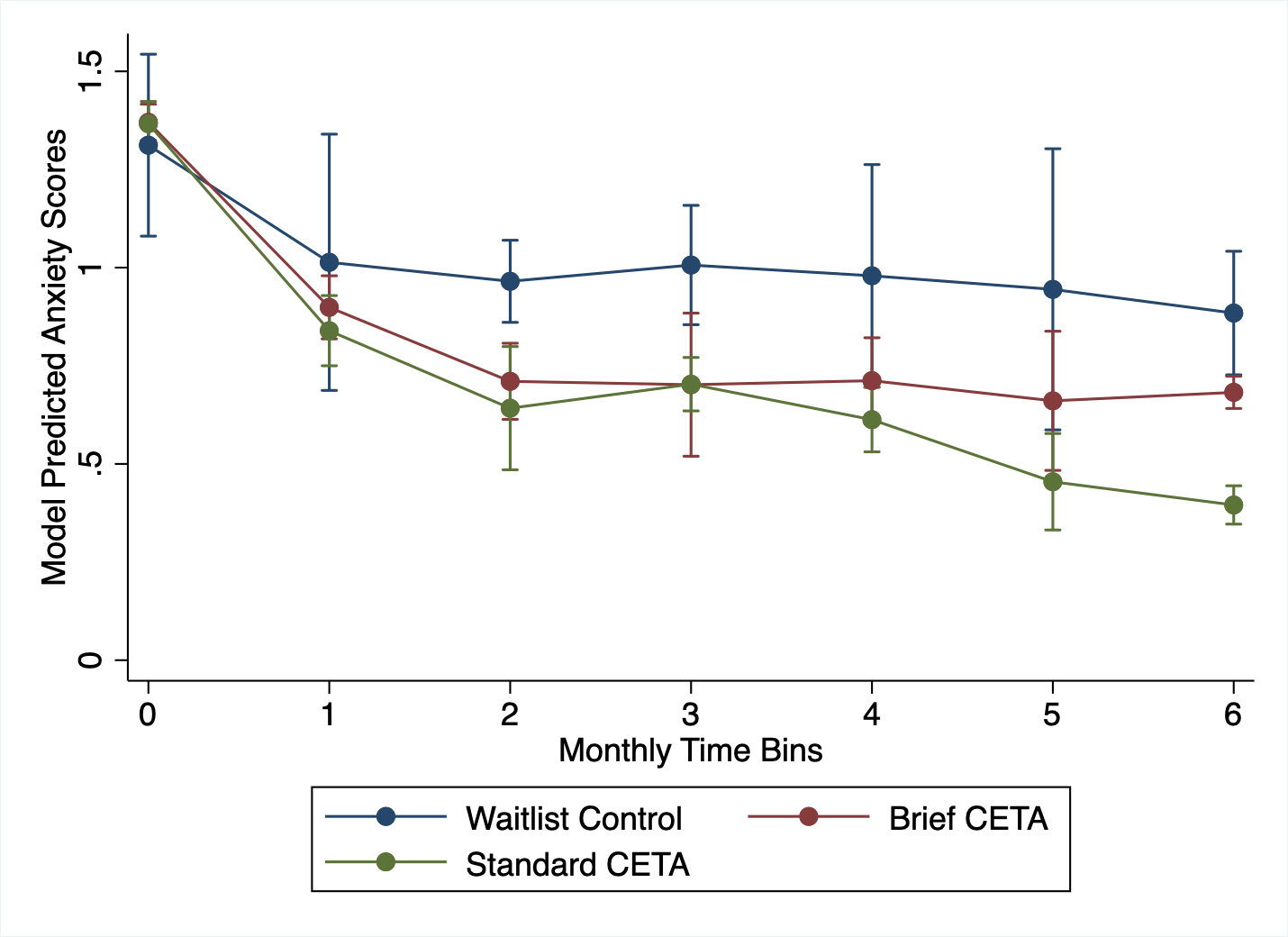


**Figure 3S.** Model predicted anxiety scores by treatment group over the six month period after baseline


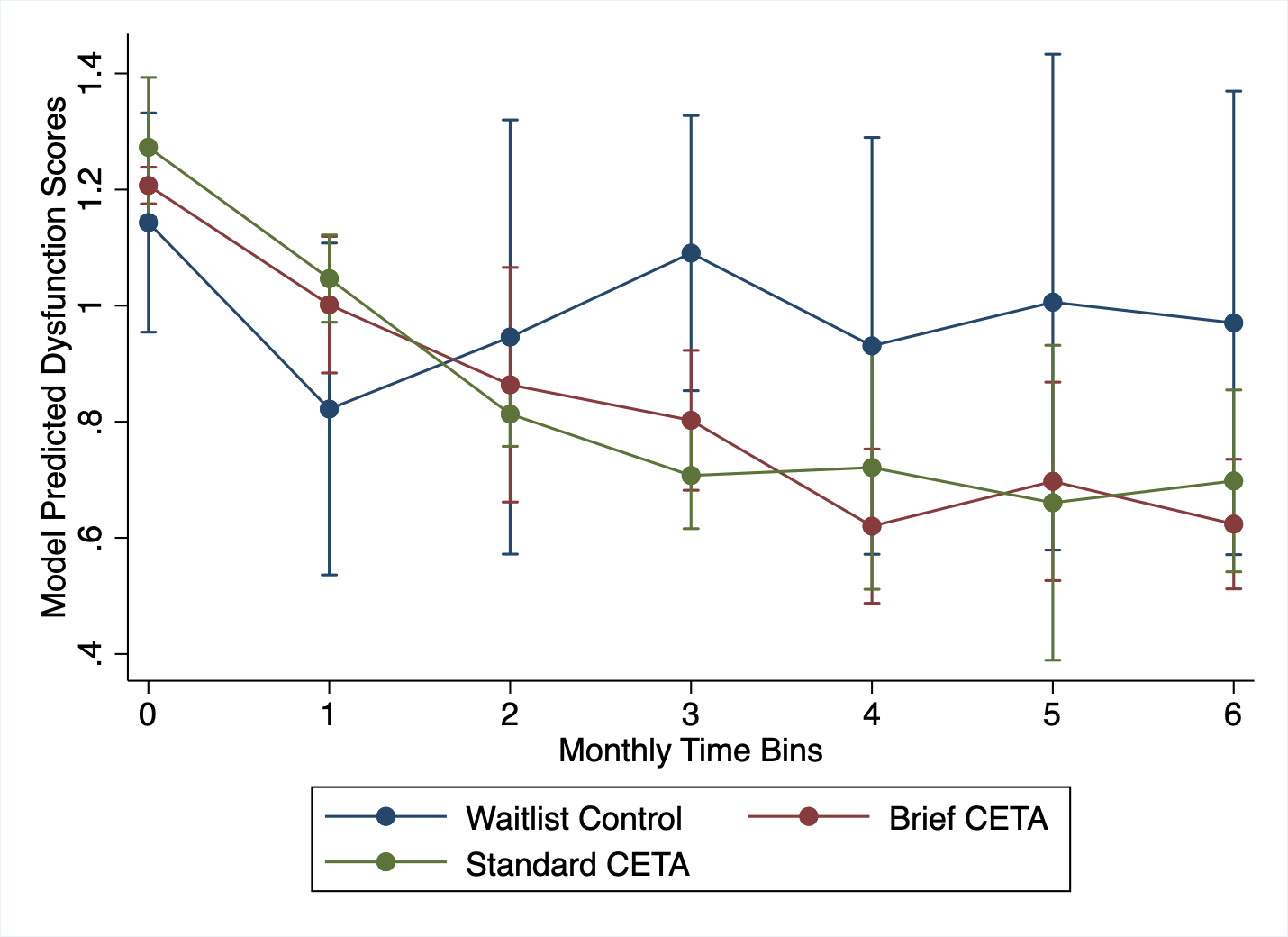


**Figure 4S.** Model predicted dysfunction scores by treatment group over the six month period after baseline
